# WeChat usage and preservation of cognitive functions in middle-aged and older Chinese adults: indications from a nationally representative survey, 2018–2020

**DOI:** 10.1186/s12889-024-19210-5

**Published:** 2024-07-04

**Authors:** Yan Zhou, KaiweiSa Abuduxukuer, Chuchu Wang, Jin Wei, Wenming Shi, Yongzhen Li, Guang Huang, Yifan Zhou, Yunfeng Zhang, Jianfeng Luo

**Affiliations:** 1 Department of Neurology, Putuo People’s Hospital, School of Medicine, Tongji University, Shanghai 200060, China; 2 Department of Pulmonary and Critical Care Medicine, Shanghai Putuo District Liqun Hospital, Shanghai, China; 3 Department of Pulmonary and Critical Care Medicine, Tongji Hospital, School of Medicine, Tongji University, No. 389 Xincun Road, Shanghai 200065, China; 4 NHC Key Laboratory of Health Technology Assessment, Fudan University, Shanghai, China; 5 Key Laboratory of Public Health Safety of Ministry of Education, Fudan University, Shanghai, China; 6 Department of Biostatistics, School of Public Health, Fudan University, Shanghai, China; 7 Department of Ophthalmology, Shanghai Municipal Hospital of Traditional Chinese Medicine, Shanghai University of Traditional Chinese Medicine, Shanghai, China; 8 Shanghai First Maternity and Infant Hospital, Tongji University School of Medicine, Shanghai, China; 9 Department of Nutrition and Food Hygiene, School of Public Health, Peking University, Beijing, 100191 China; 10 School of Public Health, Bengbu Medical University, Anhui, China; 11 Department of Ophthalmology, Shanghai Tenth People’s Hospital, School of Medicine, Tongji University, Shanghai, China

**Keywords:** China health and retirement longitudinal study, Cognitive performance, Episodic memory, Executive function, Social media, WeChat

## Abstract

**Purpose:**

To investigate the associations between the most popular social media platform WeChat usage and cognitive performance among the middle-aged and older Chinese population using data from a nationally representative survey.

**Methods:**

In total, 17,472 participants (≥ 45 years old) from the China Health and Retirement Longitudinal Study (CHARLS, Wave 4, 2018) were analyzed. Cognitive performance including episodic memory and executive function was assessed using Mini-Mental Status Examination (MMSE). Other confounding variables included socio-economic characteristics, medical status, and lifestyle-related information. Multiple linear regression models were used to test the association between cognitive performance and WeChat usage by introducing covariates hierarchically. Subgroup analyses of age and gender were conducted to estimate the robustness of the primary findings.

**Results:**

After adjusting for multiple confounders across all linear models, WeChat usage is significantly associated with executive function, episodic memory, and global cognitive performance (all p values<0.05). Such results remained robust in subgroup analyses, stratified by age and gender, and also verified according to longitudinal analyses. Compared to ‘Chat-only’ users who only used WeChat for online interpersonal communication, further usage of WeChat functions such as using ‘Moments’ appeared to be significantly associated with better cognitive performance, especially for episodic memory.

**Conclusion:**

Social media usage is significantly and positively associated with better cognitive performance among the middle-aged and older Chinese population. Along with point-to-point messaging, using ‘Moments’ and extended social media platform functions may correlate to better cognitive performance.

**Supplementary Information:**

The online version contains supplementary material available at 10.1186/s12889-024-19210-5.

## Introduction

As a consequence of global aging, cognitive impairment in the aging population has become one major public health and social concern. Around 55 million people are currently living with cognitive impairment or dementia, and this number could increase to 152 million by 2050 according to proper estimation [1]. Many countries have identified the preservation of cognitive function in the aging population as a public health priority. More than one-third of dementia cases could be prevented or intervened by precautionary measures that address modifiable risk factors such as a healthier diet or more physical activities [2, 3]. Therefore, the identification of associated factors for the preservation of cognitive function in the aging population has become a central topic of investigation.

Social media refers to Internet-based communication platforms for interpersonal communications among its users [4]. With the development and popularization of Internet technologies, using social media has become an integral part of one’s daily activities. Especially, during the past decade, the striking increase in the number of older users has become a conspicuous aspect of the social media markets [5]. Recent studies have indicated social media as a new dimension affecting people’s mental health status [6], owing to increased interpersonal communications and consequently enhanced social support [7] and self-perceived emotional support [8]. Using social media was also found to be associated with better memory performance and lower levels of depression in the older population [9]. Several small sample pilot studies have reported popular social media such as Facebook and Twitter as interventions to benefit cognitive performance in the senior population [10, 11]. However, report of real-world data with a large-scale sample is still lacking regarding the associations between social media usage and the preservation of cognitive function in aging population. Moreover, there is limited literature on this issue from developing countries.

China is one of the most populous developing countries, which is also facing severe mental health problems including cognition impairment among its huge scale of aging population. A recent report on dementia and mild cognitive impairment (MCI) among elderly Chinese from a national level showed a prevalence of 6.9% for dementia, which indicates 15.07 million Chinese aging population, and a prevalence of 15.5% for MCI, which reflects 38.77 million senior adults in China [12]. By 2050, it is estimated that China’s population with dementia would reach 10 million, which would inevitably create an overwhelming burden for individuals, their families, and the entire society [13, 14]. On the other hand, with the continuous development of China’s Internet industry, the number of older Internet users in China has also been increasing. It was reported in the 49th Statistical Report on China’s Internet Development by China’s Internet Information Center in 2022, that the number of older Chinese Internet users (people over 60 years old) had reached 119 million in 2021, and over 90% of these older users used social media [15].

Similar to the popularity of Facebook and Twitter in Western countries, with over one billion domestic active users [16], WeChat holds an absolutely predominant market share of point-to-point instant messaging and multipurpose social media platform in mainland China, whose functions include, but are not limited to online interpersonal communication, and financial services such as online payment, or even public platform functions such as health check, education and supports during the COVID-19 pandemic [17]. So far, several studies have reported the impacts of WeChat use on Chinese younger users’ mental health issues, such as its suppressing effect on the negative impacts of stressful life events on life satisfaction [18], and its influence on people’s depressive mood [19]. However, to our knowledge, there is a paucity of nationwide empirical reports that dealt with the relationships between social media use (WeChat) and mental health status, especially cognitive performance, among the older Chinese population. Owing to the distinct political institutions, network management, and domestic customs, the mental status of our aging people had not received enough attention for a long time, and the impact of social media usage in this population had not been widely investigated. To address the research gap, using a large, population-based sample derived from a nationally representative survey: the China Health and Retirement Longitudinal Study (CHARLS), the current study aims to investigate the associations between WeChat usage and cognitive functions among the aging Chinese population.

## Methods

### Participants and public involvement

**Fig. 1:**
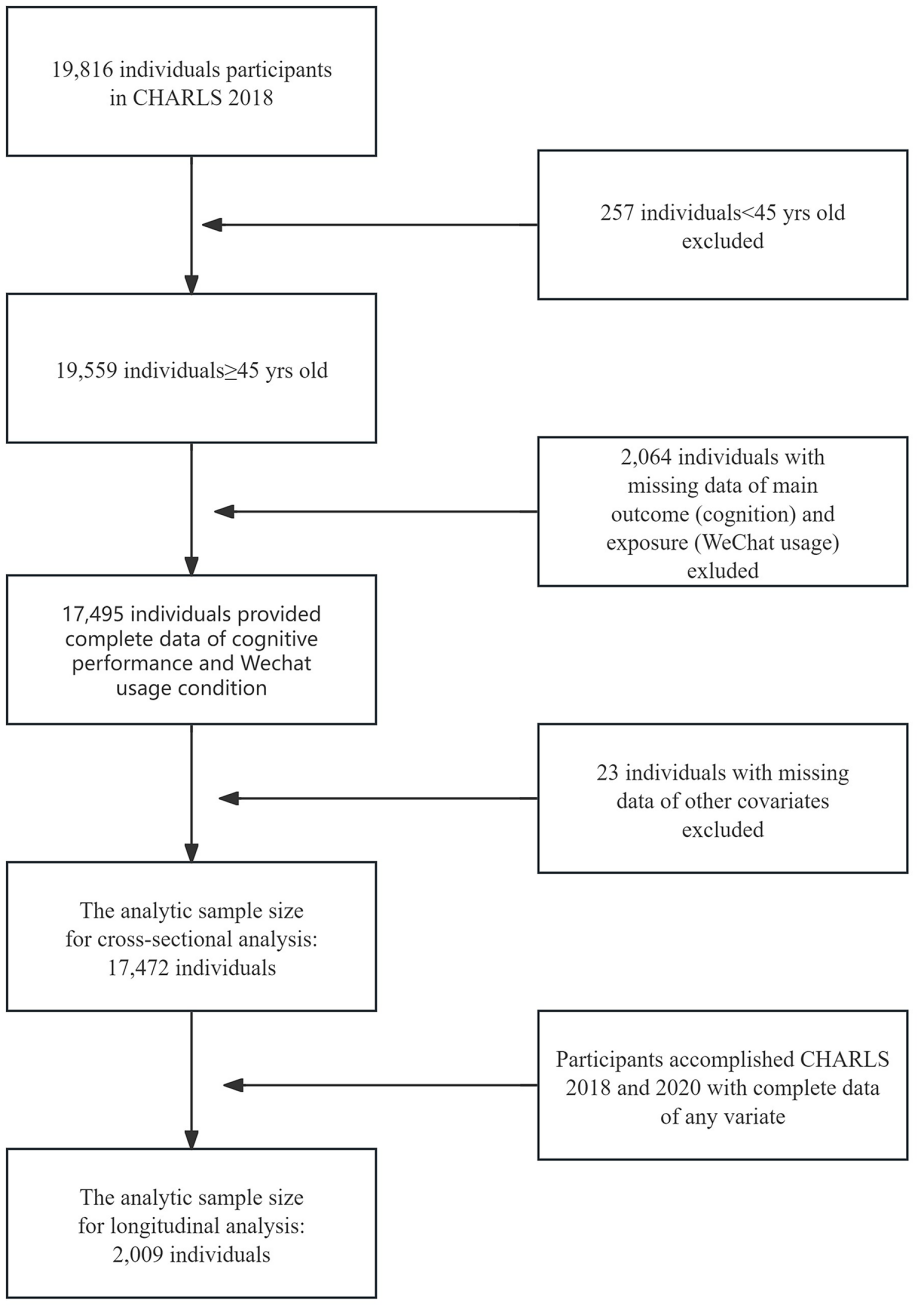
Sample screening of the present study

Data was obtained from the very first nationally representative longitudinal survey in the Chinese mainland, the China Health and Retirement Longitudinal Study (CHARLS), which enrolled sampling residents (middle-aged and older adults, aged 45 or older) from 450 villages/neighborhoods and 150 counties across 28 provinces in the Chinese mainland. CHARLS provides the most up-to-date longitudinal datasets for investigation of the health and well-being status of the middle-aged and older population in the Chinese mainland. We adopted data from CHARLS 2018 (Wave 4, 19,816 participants) for the statistical analyses in the present study. According to the purpose of the current study, participants over 45 years old were selected for analysis, and those who had missing data in WeChat usage, cognitive evaluation or any of the covariates were excluded (Fig. 1). For longitudinal analysis, participants with complete data from CHALRS 2018 and CHARLS 2020 were deemed eligible (Fig. 1).

### Measures

#### Outcome

The main outcome of this study is cognitive functions among our study sample. The evaluation of cognitive function in the CHARLS 2018 questionnaire has been described in numerous studies [20, 21]. In brief, CHARLS adopted the Chinese version of the Mini-Mental Status Examination (MMSE), which concluded similar concepts to those accepted in the American Health and Retirement Study (HRS) [22, 23]. Two core dimensions of cognitive functions were evaluated in CHARLS, including executive function and episodic memory. Executive function was evaluated from three dimensions, which included orientation, mathematical performance, and visuoconstruction, according to the Telephone Interview of Cognitive Status (TICS-10) and figure redrawing [20, 21, 24]. Episodic memory was assessed by immediate and delayed word recall [25]. The CHARLS 2020 questionnaire adopted a simplified version of MMSE, and only global cognitive function scores were available for further longitudinal study in the current research.

#### Independent variable

The independent variable in the present study was social media (WeChat) usage. Participants were asked “Do you use WeChat?”, and those who responded positively were defined as WeChat users. Then, WeChat users were asked, “Do you post WeChat Moments?”. “Moments” is a function for users to share photos or post comments among contacts, which could be regarded as further usage of the WeChat functions. We divided participants into three groups to see how social media usage exerts divergent influences on cognitive performance: [1] Those with a negative response to WeChat use were regarded as “Reference” group; [2] Those with an answer of “yes” to WeChat use but do not use “Moments” were assigned in “Contact only” group, and [3] those who further uses “Moments” function of WeChat were assigned in “Plus Moments” group.

#### Control variates

Gender was considered as a binary variate. Age was considered as a continuous variate. Marital status referred to the living status of participants with or without their spouses. Response of never married, divorced, separated, or widowed were considered as “Separated”. Participants who responded married or partnered were regarded as “Coupled”. Living area referred to places where the participant lived. Educational level indicated general socio-economic status of the respondents. Educational level was categorized into five groups (from up to low): high school or above, middle school, elementary school, less than elementary school and illiterate. Lifestyle included drinking habit and smoking status. Drinking was treated as a three-category variate that indicates the frequency of alcohol consumption: more than once a month, less than once a month, or none. Smoking status was classified as never smoked or current/former smoker. For medical history, participants were asked, “Have you ever been diagnosed with the following medical conditions?“, which includes diabetes, dyslipidemia, hypertension, cancer, lung/liver/heart/kidney/stomach or digestive diseases, stroke, emotional, neural or psychiatric problems, memory-related diseases, arthritis or rheumatism, and asthma. We defined those who reported more than 2 diseases as ‘Multi-morbidities’ [26]. Medical insurance coverage represented the approach to health support. Responders were asked, ‘Are you the policyholder/primary beneficiary of any type of health insurance?’ [27].

### Statistical analysis

Statistical analyses were performed using SAS, version 9.4 (SAS Institute, Cary, NC, USA). In this study, the primary exposure of interest was WeChat usage, while the other independent variables were selected as covariates and were reported as means ± SD or numbers(%). For comparison among the different groups according to WeChat usage statuses, one-way analysis of variance (ANOVA) or the Kruskal–Wallis test for continuous variables, and the χ^2^ test for categorical variables were used to evaluate differences. Cross-sectional analyses of associations between WeChat usage and cognitive functions were assessed using multiple linear regression models. Multi-confounders including socio-demographic factors, lifestyles, and medical conditions were hierarchically added into regression models for adjustment of their confounding impacts.

The influences of different age groups are rarely mentioned in previous related studies. Also, there seem to be explicit disparities in cognitive performance across different genders [20]. Therefore, we further performed subgroup analyses according to gender and age as sensitivity analyses.

## Results

After the exclusion of participants who were younger than 45 years old (261 participants) or with missing data, 17,472 participants from CHARLS 2018 were deemed available for the current study (Fig. 1). The participants’ distribution based on WeChat usage status was shown in Table 1, including a description of differences in socio-economic status, lifestyles, medical condition and descriptive statistics for MMSE scores among users and non-users. Comparatively, male participants have a relatively higher usage of WeChat than female participants across all age categories (Supplementary Table 1). As expected, WeChat users had much better performance in instant and delayed word recall (episodic memory) and executive function, which lead to higher global cognition scores than non-users. Compared to Non-users, WeChat users appeared to be relatively younger, more educated, and more likely to live with a partner and dwell in urban areas. WeChat users also had relatively higher alcohol consumption and insurance coverage.

**Table 1:** Characteristics of study sample from CHARLS 2018

Variables	Total	non	only wechat	wechat and moments	*P* value
**Age**	61.47 ± 9.59	62.41 ± 9.54	55.20 ± 7.42	55.23 ± 7.16	< 0.0001
**Gender**					< 0.0001
Male	8407(48.12)	7155(47.13)	300(52.36)	952(55.45)	
Female	9065(51.88)	8027(52.87)	273(47.64)	765(44.55)	
**Education**					< 0.0001
Illiterate	3526(20.18)	3488(22.97)	24(4.19)	14(0.82)	
Less than elementary school	7708(44.12)	7160(47.16)	177(30.89)	371(21.61)	
Middle school	3965(22.69)	3160(20.81)	190(33.16)	615(35.82)	
High school or vocational school	1905(10.90)	1224(8.06)	136(23.73)	545(31.74)	
college and above	368(2.11)	150(0.99)	46(8.03)	172(10.02)	
**Marital status**					< 0.0001
Coupled	13,987(80.05)	12,078(79.55)	473(82.55)	1436(83.63)	
Separated	3485(19.95)	3104(20.45)	100(17.45)	281(16.37)	
**Living area**					< 0.0001
Urban	3510(20.09)	2559(16.86)	213(37.17)	738(42.98)	
Rural	13,962(79.91)	12,623(83.14)	360(62.83)	979(57.02)	
**Drinking status**					< 0.0001
Drink more than once a month	4749(27.18)	3922(25.83)	176(30.72)	651(37.91)	
Drink but less than once a month	1340(7.67)	982(6.47)	84(14.66)	274(15.96)	
None of these	11,383(65.15)	10,278(67.70)	313(54.62)	792(46.13)	
**Smoking status**					0.6957
Yes	7119(40.75)	6171(40.65)	232(40.49)	716(41.70)	
No	10,353(59.25)	9011(59.35)	341(59.51)	1001(58.30)	
**Multi-morbidities**					0.6963
Yes	1103(6.31)	953(6.28)	41(7.16)	109(6.35)	
No	16,369(93.69)	14,229(93.72)	532(92.84)	1608(93.65)	
**Insurance covering**					0.0164
Yes	16,983(97.20)	14,737(97.07)	559(97.56)	1687(98.25)	
No	489(2.80)	445(2.93)	14(2.44)	30(1.75)	
**Executive Function**	6.52 ± 3.03	6.19 ± 3.00	8.42 ± 2.44	8.77 ± 2.21	< 0.0001
**Episodic Memory**	3.39 ± 2.40	3.11 ± 2.35	4.95 ± 1.94	5.34 ± 1.83	< 0.0001
**Global Cognitive Performance**	9.91 ± 4.82	9.30 ± 4.71	13.37 ± 3.62	14.10 ± 3.30	< 0.0001

Table 2 shows the linear regression models which indicated the potential associated factors of cognitive performance in our sample. WeChat usage, along with other factors including gender, age, educational level, living area, smoking, alcohol consumption, and medical conditions were all found to have certain associations with cognitive performance. The results of the univariate linear regression models indicated variables that could probably confound the relationships between WeChat usage and cognitive performance in multivariate models. Thus, to further clarify the correlations between WeChat usage and cognitive performance, we reanalyzed their relevance by controlling the covariates (Table 3). We noticed profound and positive associations between WeChat usage and better cognitive performance in both episodic memory and executive function even after being adjusted for various confounders across all four models (all p values<0.001, Table 3). Such correlations remained significant in subgroup analyses (stratified by gender, Table 4, and age, cut-off point: 60 years old, Table 5).

**Table 2:** Univariate linear regression analysis of independent variables and cognitive performance

	Executive Function		Episodic Memory		Global Cognitive Performance	
Variables	β(95%CI)	*P* value	β(95%CI)	*P* value	β(95%CI)	*P* value
**Gender**						
Female	Reference					
Male	1.220(1.131,1.308)	<0.0001	0.270(0.199,0.341)	<0.0001	1.490(1.349,1.631)	<0.0001
**Age**	-0.070(-0.074,-0.065)	<0.0001	-0.098(-0.101,-0.095)	<0.0001	-0.168(-0.175,-0.161)	<0.0001
**Marital status**						
Separated	Reference					
Coupled	0.925(0.812,1.036)	<0.0001	0.843(0.755,0.931)	<0.0001	1.768(1.591,1.945)	<0.0001
**Education**						
Illiterate	Reference					
Less than primary school	3.000(2.900,3.099)	<0.0001	2.016(1.935,2.097)	<0.0001	5.015(4.865,5.166)	<0.0001
Middle school	4.459(4.346,4.572)	<0.0001	3.301(3.209,3.393)	<0.0001	7.760(7.589,7.931)	<0.0001
High school or vocational school	5.105(4.966,5.245)	<0.0001	3.796(3.673,3.900)	<0.0001	8.892(8.681,9.102)	<0.0001
Collage and above	5.825(5.557,6.094)	<0.0001	4.210(3.992,4.428)	<0.0001	10.035(9.630,10.441)	<0.0001
**Living area**						
Rural area	Reference					
Urban area	1.624(1.514,1.733)	<0.0001	1.051(0.964,1.138)	<0.0001	2.675(2.501,2.849)	<0.0001
**Smoke**						
No	Reference					
Yes	0.669(0.578,0.760)	<0.0001	0.015(-0.057,0.088)	0.677	0.684(0.539,0.829)	<0.0001
**Drinking status**						
No drink	Reference					
Drink but less than once a month	1.011(0.910,1.112)	<0.0001	0.512(0.432,0.593)	<0.0001	1.523(1.362,1.684)	<0.0001
Drink more than once a month	1.180(1.010,1.349)	<0.0001	0.887(0.753,1.022)	<0.0001	2.067(1.798,2.336)	<0.0001
**Multi-morbidities**						
No	Reference					
Yes	-0.196(-0.381,-0.011)	0.0377	-0.204(-0.350,-0.057)	0.0063	-0.400(-0.693,-0.106)	0.0076
**Insurance covering**						
No	Reference					
Yes	1.685(1.414,1.957)	<0.0001	1.048(0.833,1.263)	<0.0001	2.733(2.302,3.164)	<0.0001

**Table 3:** Multivariate linear regression analysis of WeChat usage and cognitive performance

WeChat usage	Model 1^a^		Model 2^b^		Model 3^c^		Model 4^d^	
	β	95%CI	β	95%CI	β	95%CI	β	95%CI
	**Executive Function**							
Ref: NO^e^								
Chat only	1.734***	(1.493,1.975)	0.881***	(0.668,1.094)	0.867***	(0.654,1.080)	0.873***	(0.660,1.085)
Chat with moments	2.069***	(1.925,2.214)	0.911***	(0.777,1.046)	0.883***	(0.748,1.018)	0.886***	(0.751,1.021)
	**Episodic Memory**							
Ref: NO								
Chat only	1.184***	(1.001,1.368)	0.560***	(0.395,0.725)	0.544***	(0.379,0.709)	0.547***	(0.382,0.712)
Chat with moments	1.578***	(1.467,1.688)	0.724***	(0.619,0.828)	0.697***	(0.592,0.802)	0.698***	(0.593,0.803)
	**Global Cognitive Performance**							
Ref: NO								
Chat only	2.919***	(2.548,3.289)	1.441***	(1.127,1.756)	1.411***	(1.097,1.726)	1.420***	(1.106,1.733)
Chat with moments	3.647***	(3.425,3.870)	1.635***	(1.436,1.834)	1.580***	(1.381,1.780)	1.584***	(1.385,1.783)

The results of the linear regression models were expressed as partial regression coefficient (β) with 95% confidence intervals (CI). The analytic sample size was 17,472

^a^ Model 1: adjusted for demographic factors including age and gender; ^b^ Model 2: adjusted for factors in Model 1, as well as social-economic including marital status, educational level and living area; ^c^ Model 3: adjusted for factors in Model 2, as well as lifestyle factors including smoking status and alcohol consumption; ^d^ Model 4: adjusted for factors in Model 3, as well as medical condition including multi-morbidities and insurance covering

*p < 0.05; **p < 0.01; ***p < 0.001

^e^ No: do not use WeChat

**Table 4:** Multivariate linear regression analysis of WeChat usage and cognitive performance, stratified by gender

WeChat usage	Model 1^a^		Model 2^b^		Model 3^c^		Model 4^d^	
	β	95%CI	β	95%CI	β	95%CI	β	95%CI
	**Male**, ***n* = 8,407**							
	**Executive Function**							
Ref: NO^e^								
Chat only	1.701***	(1.380,2.022)	1.028***	(0.729,1.328)	1.005***	(0.705,1.304)	1.010***	(0.711,1.309)
Chat with moments	1.714***	(1.525,1.903)	0.932***	(0.748,1.115)	0.892***	(0.708,1.075)	0.895***	(0.712,1.078)
	**Episodic Memory**							
Ref: NO								
Chat only	1.146***	(0.902,1.389)	0.658***	(0.429,0.888)	0.633***	(0.404,0.863)	0.638***	(0.409,0.868)
Chat with moments	1.309***	(1.165,1.452)	0.734***	(0.593,0.875)	0.703***	(0.562,0.845)	0.705***	(0.563,0.846)
	**Global Cognitive Performance**							
Ref: NO								
Chat only	2.846***	(2.363,3.330)	1.686***	(1.248,2.124)	1.638***	(1.200,2.076)	1.648***	(1.212,2.085)
Chat with moments	3.023***	(2.738,3.307)	1.666***	(1.397,1.934)	1.595***	(1.327,1.864)	1.600***	(1.332,1.868)
	**Female**, ***n* = 9,065**							
	**Executive Function**							
Ref: NO								
Chat only	1.788***	(1.429,2.147)	0.726***	(0.424,1.029)	0.721***	(0.418,1.023)	0.725***	(0.423,1.027)
Chat with moments	2.545***	(2.324,2.765)	0.879***	(0.680,1.078)	0.869***	(0.669,1.070)	0.871***	(0.671,1.071)
	**Episodic Memory**							
Ref: NO								
Chat only	1.233***	(0.959,1.508)	0.453***	(0.216,0.690)	0.442***	(0.205,0.679)	0.443***	(0.206,0.680)
Chat with moments	1.919***	(1.751,2.088)	0.677***	(0.520,0.833)	0.659***	(0.501,0.816)	0.658***	(0.500,0.815)
	**Global Cognitive Performance**							
Ref: NO								
Chat only	3.021***	(2.461,3.582)	1.180***	(0.730,1.629)	1.162***	(0.712,1.612)	1.168***	(0.719,1.618)
Chat with moments	4.464***	(4.120,4.807)	1.556***	(1.260,1.851)	1.528***	(1.230,1.826)	1.528***	(1.230,1.826)

The results of the linear regression models were expressed as partial regression coefficient (β) with 95% confidence intervals (CI).

^a^ Model 1: adjusted for demographic factors including age; ^b^ Model 2: adjusted for factors in Model 1, as well as social-economic including marital status, educational level and living area; ^c^ Model 3: adjusted for factors in Model 2, as well as lifestyle factors including smoking status and alcohol consumption; ^d^ Model 4: adjusted for factors in Model 3, as well as medical condition including multi-morbidities and insurance covering

*p < 0.05; **p < 0.01; ***p < 0.001

^e^ No: do not use WeChat

**Table 5:** Multivariate linear regression analysis of WeChat usage and cognitive performance, stratified by age

WeChat usage	Model 1^a^		Model 2^b^		Model 3^c^		Model 4^d^	
	β	95%CI	β	95%CI	β	95%CI	β	95%CI
	**Age < 60,** ***n*** ** = 7,841**							
	**Executive Function**							
Ref: NO^e^								
Chat only	1.550***	(1.276,1.824)	0.856***	(0.610,1.102)	0.833***	(0.587,1.080)	0.839***	(0.592,1.085)
Chat with moments	1.927***	(1.763,2.091)	0.941***	(0.783,1.098)	0.908***	(0.750,1.066)	0.905***	(0.747,1.063)
	**Episodic Memory**							
Ref: NO								
Chat only	1.089***	(0.867,1.311)	0.587***	(0.385,0.789)	0.572***	(0.369,0.775)	0.577***	(0.374,0.779)
Chat with moments	1.500***	(1.368,1.633)	0.783***	(0.654,0.911)	0.760***	(0.630,0.889)	0.758***	(0.629,0.888)
	**Global Cognitive Performance**							
Ref: NO								
Chat only	2.639***	(2.209,3.069)	1.443***	(1.072,1.814)	1.405***	(1.034,1.777)	1.415***	(1.044,1.786)
Chat with moments	3.427***	(3.172,3.683)	1.723***	(1.487,1.960)	1.668***	(1.430,1.906)	1.664***	(1.426,1.901)
	**Age ≥ 60,** ***n*** ** = 9,631**							
	**Executive Function**							
Ref: NO								
Chat only	2.729***	(2.251,3.207)	1.255***	(0.839,1.671)	1.247***	(0.831,1.662)	1.237***	(0.822,1.651)
Chat with moments	2.921***	(2.637,3.205)	1.124***	(0.867,1.381)	1.094***	(0.837,1.352)	1.103***	(0.846,1.360)
	**Episodic Memory**							
Ref: NO								
Chat only	1.816***	(1.466,2.166)	0.749***	(0.439,1.059)	0.737***	(0.427,1.047)	0.732***	(0.423,1.042)
Chat with moments	2.103***	(1.894,2.312)	0.778***	(0.586,0.971)	0.739***	(0.546,0.931)	0.741***	(0.549,0.934)
	**Global Cognitive Performance**							
Ref: NO								
Chat only	4.545***	(3.814,5.276)	2.004***	(1.395,2.613)	1.983***	(1.375,2.592)	1.969***	(1.362,2.577)
Chat with moments	5.024***	(4.590,5.458)	1.902***	(1.526,2.279)	1.833***	(1.456,2.210)	1.844***	(1.468,2.220)

The results of the linear regression models were expressed as partial regression coefficient (β) with 95% confidence intervals (CI).

^a^ Model 1: adjusted for demographic factors including gender; ^b^ Model 2: adjusted for factors in Model 1, as well as social-economic including marital status, educational level and living area; ^c^ Model 3: adjusted for factors in Model 2, as well as lifestyle factors including smoking status and alcohol consumption; ^d^ Model 4: adjusted for factors in Model 3, as well as medical condition including multi-morbidities and insurance covering

*p < 0.05; **p < 0.01; ***p < 0.001

^e^ No: do not use WeChat

Compared to the 573 Chat-only users, using ‘Moments’ appeared to be significantly associated with better episodic memory, executive function, and global cognitive performance after being adjusted for age and gender among ‘Plus Moments’ users (Table 6). And the associations between using ‘Moments’ and episodic memory remained significant even after being adjusted for various confounders across all four models (all p values<0.05, Table 6).

**Table 6:** Further usage of extended function of WeChat platform and cognitive performance

WeChat usage	Model 1^a^		Model 2^b^		Model 3^c^		Model 4^d^	
	β	95%CI	β	95%CI	β	95%CI	β	95%CI
	**Executive Function**							
Ref: Chat only								
Chat with moments	0.335**	(0.121,0.549)	0.059	(-0.144,0.263)	0.060	(-0.143,0.264)	0.056	(-0.148,0.260)
	**Episodic Memory**							
Ref: Chat only								
Chat with moments	0.396***	(0.224,0.569)	0.186*	(0.021,0.351)	0.180*	(0.015,0.345)	0.175*	(0.011,0.340)
	**Global Cognitive Performance**							
Ref: Chat only								
Chat with moments	0.731***	(0.412,1.051)	0.245	(-0.051,0.541)	0.240	(-0.056,0.537)	0.232	(-0.064,0.528)

The results of the linear regression models were expressed as partial regression coefficient (β) with 95% confidence intervals (CI). The analytic sample size was 2,290

^a^ Model 1: adjusted for demographic factors including age and gender; ^b^ Model 2: adjusted for factors in Model 1, as well as social-economic including marital status, educational level and living area; ^c^ Model 3: adjusted for factors in Model 2, as well as lifestyle factors including smoking status and alcohol consumption; ^d^ Model 4: adjusted for factors in Model 3, as well as medical condition including multi-morbidities and insurance covering

*p < 0.05; **p < 0.01; ***p < 0.001

2,009 participants who accomplished the follow-up interview in CHARLS 2020 with complete data of any covariates were enrolled in the longitudinal analysis. The cross-sectional associations between WeChat usage and cognitive performance were further verified according to longitudinal analyses (users versus non-users, all p values<0.05, Table 7). However, using ‘Moments’ did not appear to be significantly associated with better global cognitive performance than ‘Chat only’ after being adjusted for covariates (Table 7).

**Table 7:** Longitudinal linear regression analysis of WeChat usage, moments usage and global cognitive function

Wechat usage	Model 1		Model 2		Model 3		Model 4	
	**Global Cognitive Performance**							
Ref: Non-users	β	95%CI	β	95%CI	β	95%CI	β	95%CI
Chat only	3.633***	(3.029,4.237)	1.496***	(0.968,2.024)	1.467***	(0.938,1.995)	1.475***	(0.948,2.003)
Chat with moments	4.609***	(4.255,4.963)	1.752***	(1.427,2.077)	1.717***	(1.390,2.043)	1.720***	(1.394,2.046)
	**Global Cognitive Performance**							
Ref: Chat only	β	95%CI	β	95%CI	β	95%CI	β	95%CI
Chat with moments	1.044***	(0.580,1.508)	0.354	(-0.071,0.779)	0.350	(-0.076,0.777)	0.355	(-0.071,0.781)

The results of the linear models regression were expressed as partial regression coefficient (β) with 95% confidence intervals (CI). The sample size was 2,009 for a binary dependent variable

Model 1: adjusted for demorgraphic factors including age and gender; Model 2: adjusted for factors in Model 1, as well as social-economic including marital status, educational level and living area; Model 3: adjusted for factors in Model 2, as well as life style factors including smoking status and alcohol consumption; Model 4: adjusted for factors in Model 3, as well as medical condition including multi-morbidities and insurance covering

*p < 0.05; **p < 0.01; ***p < 0.001

## Discussion

To our knowledge, the present study is the first to investigate the social media (WeChat) usage condition and its associations with the cognitive performance of the middle-aged and older population in China. Although only the most popular social media platform in mainland China, WeChat, was adopted in CHARLS, which means the actual social media usage rate in the aging Chinese population might be higher than discussed in the current study. Nevertheless, with 1.2 billion monthly active users and over 100 million aging users, WeChat is the most influential and popular domestic social media platform in China. Therefore, we considered that WeChat could be regarded as the representative to investigate the associations between social media usage and cognitive functions among the Chinese population in the current study.

### Executive function

The present study is the first to report correlations between social media usage and executive functions in the middle-aged and older Chinese population. Although extended applications such as online payment, financial management, and e-commerce were gradually integrated into WeChat, its core function is still interpersonal online communication. And WeChat use by older Chinese adults is specific, with 85% using WeChat for social functions, according to data from the Tencent Research Institute [28]. Therefore, we proposed that the associations between WeChat usage and better executive functions might be explained by promoted interpersonal communications. Such premise could be supported by research by Khoo et al., who demonstrated that middle-aged and older people’s usage of social media for social connection as a useful medium that protects against age-related cognitive decline in executive functions, according to results from their equation modeling analysis of a nationally representative cohort dataset, MIDUS Refresher Survey and Cognitive Project from the United States [29]. Social media could serve as a channel for senior users to stay in close contact with their family and acquaintances [30]. Meta-analytical studies suggest that greater social activities are positively associated with executive functions and global cognition in the older population [31].

### Episodic memory

Episodic memory, as a holistic measure, can reflect the outcome of memory changes [32]. Changes in memory performance during the course of aging could be influenced by behavioral factors [33]. Online social communications demand the users read, think, interact, and learn new things all at once [34], which would also require and enhance one’s memory function. Consistent with the abundant literature, which reported the positive correlations between social media usage and memory performance [10, 35], we also revealed that WeChat usage could have a positive impact on episodic memory among the middle-aged and older Chinese population. Social media usage could yield a variety of beneficial impacts, such as more convenient access to information [36], promoted connectedness and diminished social isolation [37, 38], increased social support [39], and stimulated cognitive activities [34], all of which have been proven to be protective for memory reservation in the aging population. Although the positive association between social media usage and memory function has been reported, changes in memory performance during the course of aging are the consequence of complex interactions among organic, neural, psychological, and behavioral factors [33]. Thus, the underlying mechanisms are yet to be discovered. Future studies may focus on the neurological basis of correlations between social media usage and memory performance [40].

On the other hand, the present study is also the first to reveal empirical evidence on the mnemonic consequences of further social media usage for personal experiences, such as using ‘Moments’ among the middle-aged and older Chinese population. Along with online social communication, over 65% of aging Chinese WeChat users used further functions of WeChat such as posting Moments [28]. We noticed that compared to the ‘Chat only’ users, senior WeChat users who also used ‘Moments’ appeared to have a higher level of episodic memory (Table 1). Compared to using ‘Chat only’, using ‘Moments’ was significantly and positively associated with better episodic memory (Table 6, all p values<0.05). Further usage of social media including sharing personal memories may afford several mnemonic advantages, which may be conducive to memory function. Posting personal events online provide opportunities for rehearsal and meaning-making of the bygone that facilitate memory retention [41]. Such rehearsal and social sharing of autobiographical memory literature contribute to remembering personal experiences [42]. On the hand, the ‘Moments’ platform is designed to allow users to engage with content by commenting on it (with text, emojis, and actions such as “liking” or sharing). Such engagement promotes people’s ability to later recognize related content [43].

### Subgroup analysis

In the present study, the proportion of senior WeChat users is less than 30%, and such proportion rationally decreased with age. Considerably, the proportion of senior WeChat users in China is lower than the social media usage rate of elderly people in the U.S. (45%) and the global average level (59.0%) [35]. And the proportion of male users is relatively higher than female users across different age categories. Such finding is consistent with a previous study on older Americans which reported that there is a gender gap among seniors in using online social networks [35]. Hence, we further performed subgroup analyses, stratified by age and gender, and we noticed that the results of the linear regression remained robust.

### Strengths and limitations

Our study contributes to the literature by expanding previous research on the impact of social media use on middle-aged and older people’s cognitive functions. To our knowledge, it is the first nationwide Chinese population-based study to provide empirical evidence of the associations between the usage of social media and cognitive performance among the aging Chinese population. CHARLS is a national study with a large sample size, therefore, findings from the current study could be generalized to the entire country. Lastly, multiple factors were included and adjusted in this study’s analyses, which could otherwise potentially confound the actual relationship between WeChat usage and cognition.

Our study is not without limitations, which require caution in interpreting the findings. First, WeChat-related items were first added to the CHARLS questionnaire in 2018, and there are several concerns of proper secondary analysis when using WeChat data from CHARLS 2020. First of all, during the Covid-19 pandemic and the strict ‘lock-down’ policies in the Chinese mainland from 2019 to 2022, millions of our senior citizens were demanded to use the social media like WeChat platform to get access to the scan codes for the nuclei acid tests for the Covid-19. Accordingly, we noticed that the proportion of senior WeChat users was less than 14% in the CHARLS 2018 from the current study. However, such proportion shot up to approximately 37% in the CHARLS 2020 (data not shown in the text, could be verified in the public CHARLS datasets). We defined WeChat exposure according to CHARLS 2018, and such exposure may be influenced in CHALRS 2020. Second, as a secondary data analysis, the selection of variables included in our model is limited to the CHARLS datasets. Third, only WeChat, the most popular social media platform in the Chinese mainland, was adopted in CHARLS and regarded as representative of various social media platforms by authors. Although WeChat is the most influential and popular domestic social media platform in China, which is also the most commonly used social media among older people in China, the actual social media usage rate in the aging Chinese population might be a little higher than assessed in the current study.

## Conclusion

Our study provides noteworthy information and explicit evidence of positive correlations between social media usage and cognitive functions among the middle-aged and older Chinese population. Along with point-to-point messaging, using Moments and extended functions of applications via social media platform may exert beneficial impacts on cognitive preservation.

### Electronic supplementary material

Below is the link to the electronic supplementary material.

Supplementary Material 1

## Data Availability

The CHARLS study data are publicly available and are open to researchers all over the world. Our study is a secondary analysis conducted by using CHARLS public data. The CHARLS dataset is accessible at http://charls.pku.edu.cn/
